# Developmentally regulated promoter-switch transcriptionally controls *Runx1 *function during embryonic hematopoiesis

**DOI:** 10.1186/1471-213X-7-84

**Published:** 2007-07-12

**Authors:** Amir Pozner, Joseph Lotem, Cuiying Xiao, Dalia Goldenberg, Ori Brenner, Varda Negreanu, Ditsa Levanon, Yoram Groner

**Affiliations:** 1 Department of Molecular Genetics, The Weizmann Institute of Science, Rehovot, 76100, Israel; 2 Department of Veterinary Resources, The Weizmann Institute of Science, Rehovot, 76100, Israel; 3 Howard Hughes Medical Institute, Department of Human Genetics, University of Utah School of Medicine, Salt Lake City, Utah 84112-5331, USA; 4 Department of Medical Genetics, West China Hospital, Sichuan University; Chengdu, Sichuan, P.R. of China

## Abstract

**Background:**

Alternative promoters usage is an important paradigm in transcriptional control of mammalian gene expression. However, despite the growing interest in alternative promoters and their role in genome diversification, very little is known about how and on what occasions those promoters are differentially regulated. Runx1 transcription factor is a key regulator of early hematopoiesis and a frequent target of chromosomal translocations in acute leukemias. Mice deficient in *Runx1 *lack definitive hematopoiesis and die in mid-gestation. Expression of *Runx1 *is regulated by two functionally distinct promoters designated P1 and P2. Differential usage of these two promoters creates diversity in distribution and protein-coding potential of the mRNA transcripts. While the alternative usage of P1 and P2 likely plays an important role in *Runx1 *biology, very little is known about the function of the P1/P2 switch in mediating tissue and stage specific expression of *Runx1 *during development.

**Results:**

We employed mice bearing a hypomorphic *Runx1 *allele, with a largely diminished P2 activity, to investigate the biological role of alternative P1/P2 usage. Mice homozygous for the hypomorphic allele developed to term, but died within a few days after birth. During embryogenesis the P1/P2 activity is spatially and temporally modulated. P2 activity is required in early hematopoiesis and when attenuated, development of liver hematopoietic progenitor cells (HPC) was impaired. Early thymus development and thymopoiesis were also abrogated as reflected by thymic hypocellularity and loss of corticomedullary demarcation. Differentiation of CD4/CD8 thymocytes was impaired and their apoptosis was enhanced due to altered expression of T-cell receptors.

**Conclusion:**

The data delineate the activity of P1 and P2 in embryogenesis and describe previously unknown functions of Runx1. The findings show unequivocally that the role of P1/P2 during development is non redundant and underscore the significance of alternative promoter usage in Runx1 biology.

## Background

The mammalian RUNX1 belongs to the *runt *domain family of transcription factors. The members of this gene family, *RUNX1, RUNX2 *and *RUNX3 *are key regulators of lineage-specific gene expression in major developmental pathways [1-3]. The three RUNX proteins recognize the same DNA-motif and regulate their target genes through interaction with a common group of transcriptional co-activators or co-repressors [4-7]. Interestingly, however, the functional overlaps are minor and each RUNX protein has a distinct subset of biological functions. Accordingly, each of the corresponding RUNX knockout (KO) mice displays a unique subset of phenotypic abnormalities [3]. This lack of functional redundancy results from a tightly regulated spatio/temporal expression mediated by an intricate transcriptional control [3,8]. For example, both *Runx1 *and *Runx3 *genes are expressed in developing dorsal root ganglia, but in different classes of sensory neurons [9-12] and both are expressed in mature T cells, but at different stages during T cell development [13-15].

In the mouse embryo, expression of Runx1 is first detected in definitive hematopoietic stem cells (HSC) and in endothelial cells at HSC emergence sites [16-18]. These occurrences correlate well with the earlier findings that homozygous disruption of *Runx1 *results in a complete absence of fetal liver hematopoiesis [19,20]. Postnatally, Runx1 is highly expressed in several hematopoietic lineages including myeloid, B- and T-lymphoid cells [2,21,22] and is required for megakaryocytic maturation [23]. In contrast to the critical role of Runx1 during embryogenesis, it is less essential for adult hematopoiesis [2], albeit conditional inactivation is associated with a number of hematopoietic abnormalities including myeloproliferation [24].

Runx1 is also expressed in a number of other tissues at specific time windows during embryogenesis [2,3]. However, due to the early (E12.5) lethality of homozygous null Runx1 mice [16,19,20], relatively little is known about the function of *Runx1 *in these tissues. In developing thymus Runx1 is more abundantly expressed in the cortex [9,14] during early stages of thymopoiesis [13]. Transgenic mice that express a dominant-negative form of *Runx1 *display defects in maturation of single positive (SP) CD4 and SP CD8 thymocytes [25]. Additionally, naive T cells from these mice exhibited increased production of IL4, lack of *GATA3 *expression and enhanced Th2 differentiation of CD4^+ ^helper-T cells [26]. Selective inactivation of *Runx1 *in T-cell progenitors demonstrated that *Runx1 *acts in double negative (DN) thymocytes to repress CD4 expression and to up-regulate CD8 as cells differentiate into the double positive (DP) thymocytes [13,27]. ES cells bearing a mutant Runx1 lacking the C-terminal repression sub-domain, failed to adequately contribute to the thymus in chimera mice [28]. In the knock-in model of this mutant, the overall number of thymocytes was significantly reduced and the thymus contained a higher proportion of SP CD4 cells [28].

We have previously shown that transcription of *Runx1 *is regulated by two distantly located promoter regions designated P1 and P2 for the distal and proximal, respectively [29]. The P2 is nested within a particularly large and evolutionary conserved CpG island [3,9], while no such CpG rich region is found in P1. The two promoters are regulated in a cell type-specific manner and respond to mitogenic stimulation [30,31]. The P1- and P2-derived primary transcripts are processed into a diverse repertoire of alternatively spliced mRNAs that are differentially expressed in various cell types and at different developmental stages [3]. The P1 and P2 mRNAs differ at their 5'-coding regions [9,31], and the resulting protein isoforms differ in their biological functions [1,32-34]. Similarly, a number of P2-derived splice-variants bear shorter reading frames that lack the transcriptional activation domain and can act in a dominant negative manner [31]. Forced expression of such isoforms results in transcriptional repression of *Runx1 *target genes [35-37], and in blocking of myeloid differentiation [36]. Hence, differential usage of P1 versus P2 has a profound effect on the repertoire and nature of Runx1 isoforms in the cell, which likely impacts on the regulation of Runx1 target genes [8]. Significantly, disruption of P1 by the 12;21 chromosomal translocation results in the most common subtype of childhood acute lymphoblastic leukemia [38].

P1- and P2-transcripts also differ in the structure and function of their 5' untranslated region (5'UTR). The P1-5'UTR is relatively short and mediates cap-dependent translation, whereas the P2-5'UTR is particularly long, contains an internal ribosome entry site (IRES) and mediates cap-independent translation [3,30]. These structurally and functionally different 5'UTRs could have a profound effect on translation efficiency of Runx1 mRNAs. Indeed, in cell lines where P2 transcription predominated, P2-5' IRES-dependent translation was more efficient than P1-5' cap-dependent translation [3,30]. IRES-dependent translation is particularly important during development, in times when cap-dependent translation in proliferating or differentiating cells is down-regulated [39]. While it is clear that the regulation of *Runx1 *expression by alternative P1/P2 usage is a potentially key paradigm in the *in vivo *regulation of Runx1, very little is known about the role of the P1/P2 switch in mediating tissue and stage specific expression of *Runx1 *during development.

To investigate the biological function of the P1/P2 switch during embryogenesis, we generated mice bearing a hypomorphic *Runx1 *allele by inserting a *neomycin *(*neo*) gene into the P2 region (P2^neo^). Mice homozygous for the P2^neo ^allele (P2^neo/neo^) displayed a markedly attenuated P2 activity. Nevertheless, these mutant mice developed to term, but newborns died within a few days after birth. We show that the P1/P2 switch is developmentally regulated and that the two promoters are alternatively used during embryogenesis. P2 mediated transcription is required during early thymopoiesis for the proper development of the thymus and for differentiation of DN and DP CD4/CD8 T cells. Attenuation of P2 activity results in impaired development of fetal liver hematopoietic precursors and in enhanced apoptosis of thymocytes due to altered expression of T-cell receptors (TCRs). The data describe previously unknown functions of Runx1, delineate the activity of P1 and P2 in embryogenesis, show unequivocally that their function during development is non redundant and underscore the significance of the two promoters in *Runx1 *biology.

## Results

### Generation of Runx1 P2^neo/neo ^mice and phenotypic analysis of mutant newborns

Null mutation in *Runx1 *results in embryonal lethality [19,20]. As P2 activity starts early in embryogenesis (Levanon D. and Groner Y. unpublished), we envisaged a scenario in which germline inactivation of P2 will also result in embryonal death. To avoid this occurrence we explored means to selectively attenuate the activity of *Runx1 *P2. We have previously demonstrated that the genomic region upstream the transcription start sites of *RUNX1 *P2 was important for regulated transcription of *RUNX1 *in various cell lines [29,30]. We thus assessed whether insertion of a *neo *cassette into this region affects P2 activity in transfected cells (See additional file 1: Fig. 1A and 1B for constructs, results and methods). Transfection experiments indicated that insertion of a *neo *cassette into the P2 region specifically attenuated P2-mediated transcription in several cell lines and encouraged us to utilize this approach for evaluating the biological activity of *Runx1 *P2 *in vivo*.

**Figure 1:**
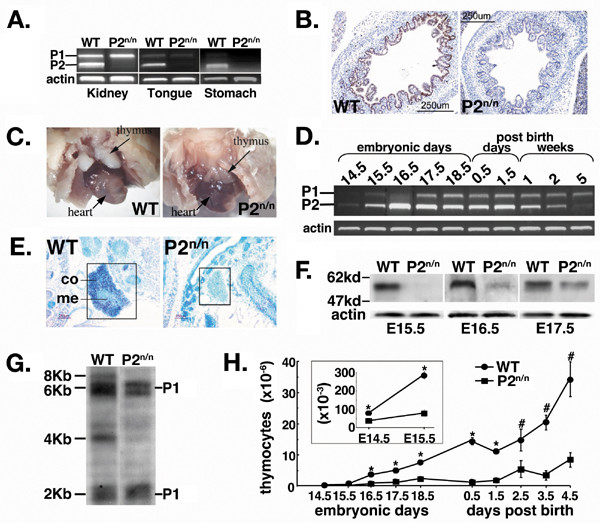
**Attenuated *Runx1 *P2 expression in P2^neo/neo ^embryos impaired thymus development**. (A) RT-PCR analysis of RNA from kidney and stomach of E16.5 WT and P2^neo/neo ^embryos, and from tongue of P1.5 WT and P2^neo/neo ^neonates. P2-mediated transcription in P2^neo/neo ^tissues diminished, whereas P1-mediated transcription was largely unaffected. (B) IHC analysis of glandular stomach of E16.5 WT (left) and P2^neo/neo ^(right) embryos. Runx1 expression is detected in epithelial cells of WT embryo, but missing in P2^neo/neo ^littermate (10× magnification). (C) Reduced size of thymic lobes in P1.5 P2^neo/neo ^mice (right) as compared to WT littermate (left). (D) RT-PCR analysis of *Runx1 *P1- and P2-mediated transcription in thymus of WT embryos, neonates and young mice. (E) Analysis of *Runx1 *expression by *In-situ *hybridization of E15.5 WT (left) and P2^neo/neo ^(right) thymic sections, using the P2-5'UTR probe. P2-derived transcripts are clearly visible in the cortex of E16.5 WT, but not of P2^neo/neo ^(10× magnification). (co) = cortex; (me) = medulla. (F) Western blot analysis of proteins extracted from thymus of E15.5–E17.5 WT and P2^neo/neo ^embryos. Runx1 proteins were not detected in E15.5 P2^neo/neo ^thymus, but gradually accumulated in E16.5 and E17.5 thymi. (G) Northern blot analysis of RNA from thymi of WT and P2^neo/neo ^newborn mice. Whereas P2-mediated transcription (the 4 Kb and 8 Kb transcripts) [30, 31], in P2^neo/neo ^thymocytes was markedly attenuated, P1-mediated transcription (the 2 Kb and 6 Kb transcripts) was apparently unaffected. (H) Reduced thymus cellularity in P2^neo/neo ^embryos and neonates. Five mice of each genotype were analyzed. The difference between the number of thymocytes from WT and P2^neo/neo ^thymus was significant at P < 0.0001 (*) and P < 0.05 (#) by Student's t test.

*Runx1 *P2 was targeted in ES cells using a construct in which the *neo *cassette was placed at the same site as in the P2^neo^-Ren construct (Additional file 1), but in a transcriptional orientation opposite to that of *Runx1*, and was flanked by lox P sites (Additional file 2A). Homologous recombination yielded ES clones (Additional file 2B), which were used to generate several *Runx1*^P2neo/+ ^chimeric males that passed on the *Runx1 *mutation through the germ line. Mating F2 heterozygotes with 129/Sv, ICR or MF1 mice generated *Runx1*^P2neo/neo ^mice with an inbred or a mixed background. Heterozygous *Runx1*^P2neo/+ ^(P2^neo/+^) mice appeared phenotypically indistinguishable from their WT littermates. However, homozygous *Runx1*^P2neo/neo ^(P2^neo/neo^) neonates were reduced in size, gained little weight and most of them died 2 to 3 days post natally (Additional file 2D).

To further characterize the phenotype/genotype relationships of the mutant mice, heterozygotes P2^neo/+ ^mice were mated with two different strains of transgenic mice expressing *Cre *driven by either *PGK *or *EIIa *promoters [40,41] (Additional file 2B). The resulting heterozygotes bearing the remaining *loxP *sequence within the P2-*XbaI *site were mated and offspring homozygous to P2-loxP were obtained at the expected Mendelian proportion (25%). Significantly, *Runx1*^P2loxP/loxP ^(P2^loxP/loxP^) mice were indistinguishable form WT littermates indicating that the early lethality phenotype of P2^neo/neo ^newborns was due to the presence of the *neo *cassette in the P2 region.

### Attenuated Runx1 expression in P2^neo/neo ^mice

The above-described phenotypic manifestation of the P2^neo/neo ^allele posed a question as to how the expression level of *Runx1 *was affected. To address this issue, *Runx1 *expression in various tissues was examined using RT-PCR, immunohistochemistry (IHC) and RNA in situ hybridization (RISH) (Fig. 1). In tissues of P2^neo/neo ^mice, such as kidney and tongue, where Runx1 expression is normally mediated by both P1 and P2, only P1-transcripts were detected (Fig. 1A). In contrast, no Runx1 expression was detected by either RT-PCR or IHC in developing gastric epithelium where only P2 is normally active (Figs. 1A and 1B). P2^neo ^is therefore a hypomorphic allele of *Runx1 *in which P2 activity is largely diminished. Interestingly, however, no developmental abnormalities were detected by macroscopic and histological examination of kidney, stomach and tongue of mutant embryos or newborns. The most obvious and consistent macroscopic abnormality was a profound reduction in size of the thymus of newborn P2^neo/neo ^mice as compared to WT (Fig. 1C).

Western blot analysis, RISH using a P2 specific probe and RT-PCR showed that during early thymogenesis (embryonal day (E) 14.5–16.5) transcription of *Runx1 *in WT thymus was predominately mediated by P2 (Fig. 1D), and that at this stage almost no Runx1 was detected in P2^neo/neo ^thymus (Figs. 1E and 1F). The P1 activity in WT thymus was switched-on between E15.5–E17.5 and persisted thereafter, whereas the activity of P2, which predominated during embryogenesis, gradually decreased after birth (Fig. 1D). Importantly, once switched-on the P1 activity was apparently unaffected in P2^neo/neo ^mice, as evidenced by northern blot analysis of *Runx1 *mRNAs in newborns (Fig. 1G). Together, the complementary results of Runx1 expression studies and morphological analyses showed that expression of Runx1 in P2^neo/neo ^mice was compromised both temporally and spatially, resulting in adverse effect on thymus development.

### Impaired thymus development in Runx1 P2^neo/neo ^mice

Thymus development in mouse embryo begins at E9.5 ensued by immigration of lymphocyte precursors into the thymic rudiment at E14.5. Development proceeds through stage specific interactions between thymocytes and thymic epithelium that are essential for the formation of thymic architecture [42]. Runx1 is highly expressed in developing thymocytes, but not in thymic epithelium [9,13,14].

**Figure 2:**
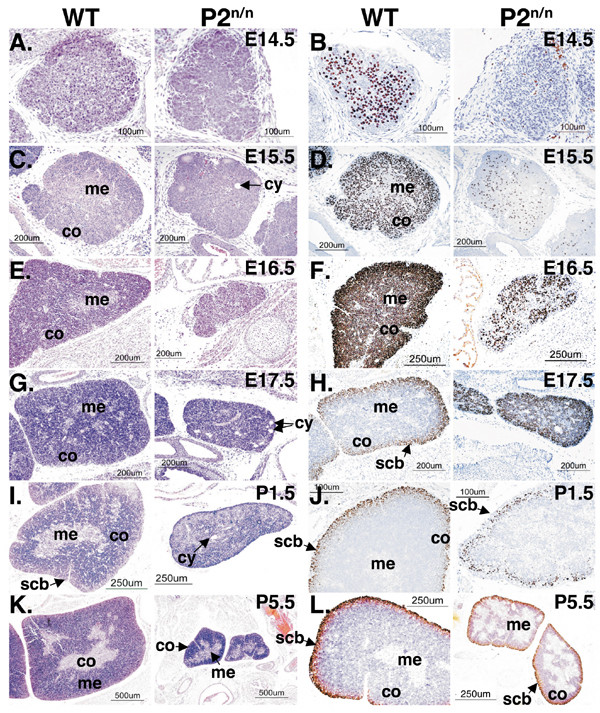
**Histological and IHC analysis of thymus development in WT and P2^neo/neo ^mice**. (A, C, E, G, I, K) Transverse sections of WT (left panels) and P2^neo/neo ^(right panels) thymic lobes stained with hematoxylin and eosin (HE). co = cortex, me = medulla, scb = sub-cortical band, cy = cyst. (B, D, F, H, J, L) Immunostaining of Runx1 in sections of WT (left panels) and P2^neo/neo ^(right panels) thymic lobes. Runx1 is detected in thymocytes, but not in thymic epithelium. A reduced number of Runx1 expressing thymocytes (that are abnormally distributed) is seen in P2^neo/neo ^thymic lobes.

Macroscopic examinations revealed that thymi of P2neo/neo neonates were about one-third of the normal size (Figs. 2I and 2K). This diminished size was caused by a profound reduction in the number of thymocytes during mutant thymus development (Fig. 1H). Histological examinations revealed no gross morphological differences between P2^neo/neo ^and WT at E14.5 and E15.5 (Figs. 2A and 2C), except for scattered cysts in P2^neo/neo ^thymi (Fig. 2C). These cysts are thought to result from accelerated epithelial turnover within centers of epithelial islands [43]. At E16.5 however, extensive histological differences between WT and P2^neo/neo ^emerged (Fig. 2E). WT thymus had a clear demarcation of cortex and medulla. WT cortex was densely cellular with small hyperchromatic thymocytes, while the medulla was paler, contained a lower number of thymocytes admixed with macrophages, epithelial cells, and antigen presenting cells (Fig. 2E). Mutant thymus, on the other hand, was much smaller, hypocellular, had a scalloped capsular surface and consisted predominantly of epithelial cells, with no evidence of an organized cortex and medulla. At E17.5 the histological defects were even more pronounced; the P2^neo/neo ^thymus still lacked corticomedullary organization and contained occasional cysts (Fig. 2G).

IHC using anti Runx1 antibodies revealed a lower number of Runx1 positive cells in E14.5 and E15.5 P2^neo/neo ^thymi compared to WT (Figs. 2B and 2D). Runx1 is mostly expressed in DN CD4^-^/CD8^- ^immature thymocytes, where it regulates CD4 expression [13,25,28]. IHC of E15.5–E16.5 WT thymus revealed that these Runx1 positive immature thymocytes were predominantly in the cortex and less in the medulla (Figs. 2D and 2F), as also shown by the RNA *in-situ *hybridization data (Fig. 1E). In P2^neo/neo ^thymus on the other hand, the fewer Runx1 positive cells were scattered randomly within the organ (Fig. 2D). At E17.5 most of Runx1 expressing DN CD4^-^/CD8^- ^thymocytes were found in the sub-cortical band (SCB), whereas the cortical, DP CD4^+^/CD8^+ ^thymocytes did not express Runx1 (Fig. 2H). In E17.5 P2^neo/neo ^thymus, many Runx1 positive cells were still in the cortex and only after birth, when the mutant thymus gradually gained WT morphology, were Runx1 expressing hyperchromatic cells seen in the SCB (Figs. 2J and 2L). But even then, the overall size of mutant thymus remained much smaller compared to WT (Figs. 2I and 2K). The data indicate that normal embryonal thymopoiesis depends on a proper P2-mediated transcription of *Runx1 *which when attenuated during the E14.5–E16.5 time period results in impaired thymus development.

### Increased apoptosis in embryonal P2^neo/neo ^thymocytes

**Figure 3:**
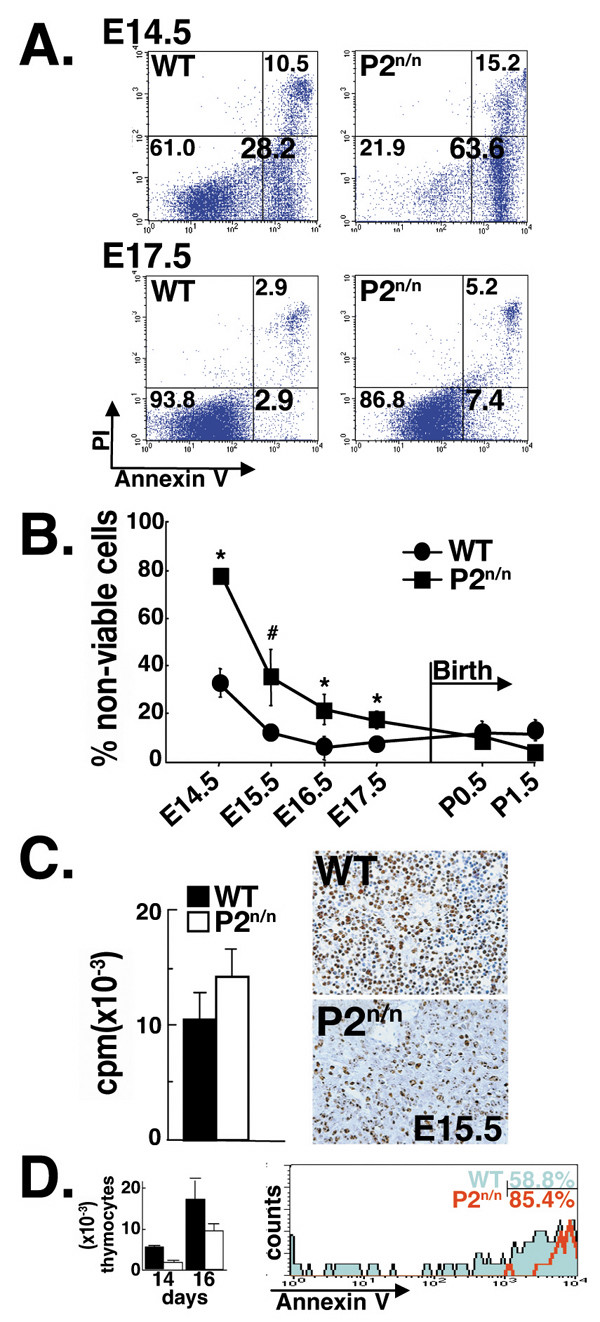
**P2^neo/neo ^thymocytes display enhanced apoptosis, but have normal cell proliferation capacity**. (A) FACS analysis of E14.5 and E17.5 WT (left panels) and P2^neo/neo ^(right panels) thymocytes stained with Annexin V and PI. FACS dot plots and percentages of cells in each quadrant are indicated. Note the >2 fold increase in the proportion of apoptotic cells (Annexin V^+^/PI^-^) and of necrotic cells (Annexin V^+^/PI^+^) in P2^neo/neo ^thymi. (B) P2^neo/neo ^thymocytes display enhanced apoptosis throughout embryonic thymopoiesis. Proportion of non-viable (apoptotic+necrotic) WT and P2^neo/neo ^cells gradually decreased during embryonic development. Nevertheless, at any given time point, P2^neo/neo ^thymui contained a higher proportion of non-viable cells compared to WT littermates. After birth the proportion of non-viable thymocytes in P2^neo/neo ^became similar to WT. At least five mice of each genotype were analyzed. The differences between WT and P2^neo/neo ^apoptotic thymocytes were significant at P < 0.001 (*) and P < 0.01 (#) by Student's t test. (C) P2^neo/neo ^thymocytes retain normal proliferation capacity. Following FACS analysis an equal number (1 × 10^4^) of E15.5 WT or P2^neo/neo ^Annexin V negative thymocytes were incubated with TPA (20 ng/ml) and ConA (5 μg/ml) for 48 h. ^3^H-thymidine was present during the last 18 h of incubation. Differences between WT and P2^neo/neo ^were statistically insignificant by Student's t test. Proliferation capability of thymocytes was further examined by immunostaining of E15.5 thymic lobes for the proliferation-associated antigen recognized by Ki-67 antibodies (Right panels). (D) Enhanced embryonal apoptosis is a cell-autonomous property of P2^neo/neo ^thymocytes. WT or P2^neo/neo ^E15.5 FL HPC were differentiated under saturating conditions in E14.5 WT FTOC for 14 or 16 days. We predetermined that ~100 FL cells (either WT or P2^neo/neo^) per lobe were sufficient to populate all available thymic lobes and therefore used 1000 FL cells per lobe. Thymocytes accumulation in FTOC populated with P2^neo/neo ^FL HPC is reduced compared to WT (left panel WT-black; P2^neo/neo^-white). Proportion of apoptotic thymocytes (Annexin V^+^) derived from FTOC populated with P2^neo/neo ^FL cells (red) was considerably higher compared to WT (light blue) (right panel).

To directly assess whether the thymic hypocellularity of P2^neo/neo ^embryos was a consequence of enhanced cell death, the proportion of apoptotic thymocytes was determined by flow cytometry. At E14.5 a much higher proportion of P2^neo/neo ^thymocytes (63% ± 5) compared to WT (28% ± 8) bound annexin V, indicating increased apoptosis among P2^neo/neo ^thymocytes (Fig. 3A). Additionally, increased proportion of necrotic cells positive to both annexin V and propidium iodid (PI), was observed in P2^neo/neo ^thymus (15% ± 3 P2^neo/neo^; 10% ± 2 WT, Fig. 3A). The enhanced cell death of E14.5 P2^neo/neo ^thymocytes led to a three-fold reduction in the proportion of viable thymocytes in mutant thymus (WT 61.0% ± 5; P2^neo/neo ^21.9% ± 3). At E16.5–17.5 the P1 promoter is switched on (Figs. 1D and 1F), expression of Runx1 in P2^neo/neo ^thymocytes increased (Figs. 2F and 2H) and recovery of thymopoiesis commenced. The end result was an increased proportion of viable P2^neo/neo ^thymocytes at E17.5 almost to WT level (Fig. 3A). The enhanced apoptosis in P2^neo/neo ^E14.5 and E15.5 thymus was also demonstrated by *in situ *TUNEL labeling and IHC of activated caspase-3 (not shown). The proportion of non-viable thymocytes at various stages of thymus development is shown in Fig. 3B. At early stages, apoptosis was significantly higher in P2^neo/neo ^fetuses compared to WT, but gradually declined concomitant with the increase in P1-mediated expression of *Runx1*.

We next asked whether the proliferative capacity of P2^neo/neo ^thymocytes was also affected. E15.5 thymocytes were isolated, analyzed by FACS and similar numbers of viable cells cultured *in-vitro *under proliferation stimulating conditions (Fig. 3C). Purified P2^neo/neo ^and WT thymocytes displayed a similar thymidine incorporation rate, indicating that the ability of thymocytes to proliferate was hardly affected by the attenuated expression of Runx1 during early thymopoiesis. This conclusion was supported by immunostaining of thymocytes with the cell proliferation-associated antigen Ki-67. In E15.5 P2^neo/neo ^thymus the number of Ki-67 positive cells was lower than in WT (Fig. 3C), reflecting the hypocellularity of mutant thymus (Fig. 2D). Nevertheless, significant numbers of P2^neo/neo ^thymocytes did express Ki-67, indicating normal progression through the cell cycle. Together, these results show that P2^neo/neo ^thymocytes possess proliferation and cell cycle progression capabilities similar to WT, but attenuating Runx1 expression during early developmental stages rendered thymocytes more susceptible to apoptosis, leading to thymic hypocellularity.

To address whether the increased apoptosis of P2^neo/neo ^thymocytes was due to loss of a cell-autonomous function of Runx1, we examined the capacity of P2^neo/neo ^thymocytes to populate fetal thymic organ culture (FTOC). E14.5 WT thymic lobes were reconstituted, at a saturating cell number, with either WT or P2^neo/neo ^E15.5 fetal liver (FL) cells that contain hematopoietic progenitor cells (HPC). At FTOC day 14 and 16, cells were extracted out of the lobes and analyzed by FACS for Annexin V binding. P2^neo/neo ^HPC gave rise to a significantly lower number of FTOC cells compared to WT and most P2^neo/neo ^derived cells were Annexin V^high ^(Fig. 3D). These data further demonstrated the intrinsic requirement for P2-mediated expression of *Runx1 *during early embryonal thymopoiesis.

### The enhanced apoptosis of P2^neo/neo ^thymocytes is associated with increased expression of TCRβ/TCRγδ

**Figure 4:**
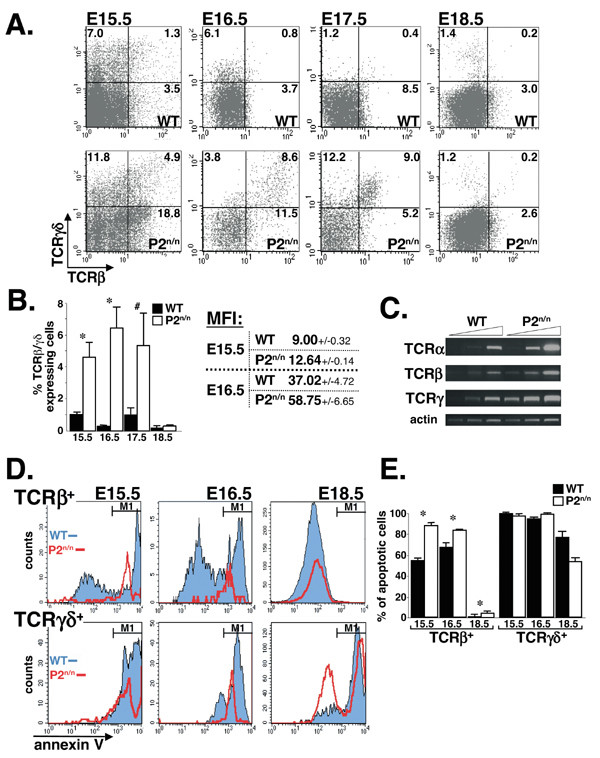
**The propensity of P2^neo/neo ^thymocytes to undergo apoptosis is associated with elevated expression of TCRβ and TCRγδ**. (A and B) Expression of TCRβ and TCRγδ on WT and P2^neo/neo ^embryonal thymocytes. (A) Shown are FACS analysis dot plots of E15.5 to E18.5 WT and P2^neo/neo ^thymocytes indicating the percentages of cells in each quadrant. Histograms in (B) show the average ± S.E. of TCRβ/TCRγδ proportions of WT and P2^neo/neo ^thymocytes in at least three experiments at each developmental stage. The difference between WT and P2^neo/neo ^was significant at P < 0.005 (*) and P < 0.05 (#) by Student's t test. While at E15.5 to E17.5 the proportion of TCRβ/TCRγδ expressing thymocytes was much higher in P2^neo/neo ^compared to WT, at E18.5 the distribution of P2^neo/neo ^thymocytes resumed normal pattern. The mean fluorescence intensity (MFI) (B right) of E15.5 and E16.5 TCRβ^+ ^P2^neo/neo ^thymocytes was significantly higher than WT (p < 0.005; by Student's t test). (C) Expression of TCRβ/TCRγδ in P2^neo/neo ^thymocytes is transcriptionally upregulated. RT-PCR analysis of RNA derived from WT and P2^neo/neo ^E16.5 thymocytes using primers specific for the pre-TCRα and the constant regions of TCRβ and TCRγ transcripts. Increased number of PCR cycles shows elevated steady-state levels of mRNAs in P2^neo/neo ^thymocytes compared to WT. (D and E) Enhanced apoptosis of P2^neo/neo ^thymocytes is associated with elevated expression of TCRβ/TCRγδ. (D) Histograms demonstrating the proportion of apoptotic cells among TCRβ^+ ^or TCRgδ^+ ^thymocytes. TCRβ/TCRγδ positive WT thymocytes (blue) are divided into two main populations, apoptotic (M1) and non-apoptotic, according to level of Annexin V (except for E15.5 TCRγδ^+ ^where all cells are apoptotic). Most of TCRβ/TCRγδ positive P2^neo/neo ^thymocytes (red), are at the M1 (Annexin V^high^) apoptotic subset. (E) Proportion of apoptotic thymocytes among TCRβ/TCRγδ positive E15.5, E16.5 and E18.5 thymocytes. Bar*s *represent the average ± S.E. of at least three independent experiments for each time point using different mice. The differences between WT and P2^neo/neo ^in number of TCRβ^+ ^apoptotic thymocytes were significant at P < 0.01 (*) by Student's t test.

The above results raised the question of how does attenuated expression of *Runx1 *cause the increased apoptosis of P2^neo/neo ^thymocytes. The majority of immature thymocytes with a non-productive rearrangement of TCRβ gene die through Fas/Fas ligand-mediated apoptosis [44]. Only a small subset of immature thymocytes with productive TCRβ or γδ rearrangement are selected for further maturation associated with rapid proliferation [45-48]. Interestingly, the percentage of TCRβ/TCRγδ expressing thymocytes was significantly higher in E14.5 to E17.5 P2^neo/neo ^fetuses compared to WT (Figs. 4A and 4B), as was the mean-fluorescence-intensity (MFI) level of TCRβ^+ ^thymocytes (Fig. 4B). These data indicate an enhanced TCRβ/γδ expression on P2^neo/neo ^thymocytes compared to WT during the early stages of embryonal thymopoiesis.

Runx1 acts in transfected cells as a negative regulator of TCRβ transcription [4]. It is thus possible that the diminished Runx1 expression between E14.5 to E16.5 caused de-repression of TCR transcription in P2^neo/neo ^thymocytes, leading to the increased level of TCRβ/γδ. Supporting this assumption are data demonstrating that upregulation of TCRβ/γδ in P2^neo/neo ^thymocytes occurred at the transcriptional level (Fig. 4C), and that down regulation to WT levels occurred at E18.5 (Fig. 4A and 4B), upon turning-on of P1 (Fig. 1).

We next addressed whether increased expression of TCRβ/γδ in P2^neo/neo ^thymocytes was associated with the increased susceptibility to apoptosis of the mutant cells. Annexin V staining was monitored in fractionated TCRβ^+ ^or TCRγδ^+ ^thymocytes (Figure 4D). In E15.5 and E16.5 embryos 50–65% of WT and 80–90% of P2^neo/neo ^TCRβ^+ ^thymocytes were apoptotic as were ~95% of both WT and P2^neo/neo ^TCRγδ^+ ^thymocytes (Figs. 4D and 4E). Additionally, at these developmental stages a significantly higher proportion of P2^neo/neo ^thymocytes express the TCRs (Figs. 4A and 4B). Thus, the enhanced apoptosis in P2^neo/neo ^compared to WT thymus (Figures 3A and 3B), was closely associated with an increase in TCRβ/γδ expression. Of note, MFI of Annexin V^+ ^apoptotic P2^neo/neo ^thymocytes, was lower compared to the WT (Figure 4D), presumably due to a lower level of cell surface phosphatidyl-serine in P2^neo/neo ^cells.

Together, the complementary outcome of these experiments indicates that Runx1 functions *in vivo *as a negative regulator of TCR expression. When the temporal expression of Runx1 is attenuated, levels of TCRβ^+^/TCRγδ^+ ^thymocytes increase, which may lead to enhanced cell death. When P1 is turned-on, restoring Runx1 expression in P2^neo/neo ^cells, apoptosis in mutant thymocytes assumes normal levels.

### Impaired thymocyte differentiation in P2^neo/neo ^embryos

**Figure 5:**
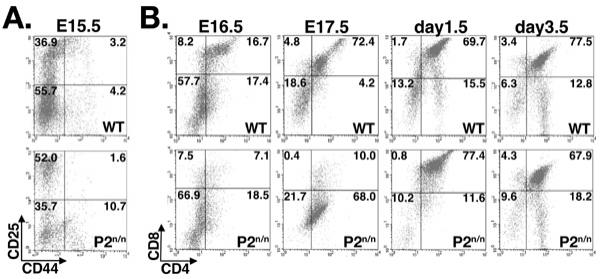
**P2-mediated expression of *Runx1 *is required for embryonal thymopoiesis**. (A) Distribution of CD44/CD25 expressing thymocytes of E15.5 WT and P2^neo/neo ^embryos. Representative data of five different experiments are shown as FACS dot plots; percentages of cells in each quadrant are indicated. (B) Distribution of embryonic and neonatal CD4/CD8 thymocytes. WT and P2^neo/neo ^thymocytes of E16.5 and E17.5 embryos and day 1.5 and 3.5 neonates were analyzed. Representative data of five different experiments are shown as FACS dot plots; percentages of cells in each quadrant are indicated.

We next employed flow cytometry to study thymocyte differentiation in P2^neo/neo ^embryos and neonates. First we examined the CD4/CD8 double negative (DN) population at E15.5 when nearly 100% of thymocytes are DN. The DN population is divided into four sub-groups, DN1 to DN4, according to the expression of CD25 and CD44 surface markers. Compared to WT, P2^neo/neo ^embryos displayed an increase in the proportion of DN3 (CD25^+^/CD44^-^) thymocytes and a corresponding reduction in DN4 cells (CD25^-^/CD44^-^) (Fig. 5A), resulting in a significant increase in the ratio DN3/DN4 among P2^neo/neo ^thymocytes (WT DN3/DN4 = 0.65 ± 0.15 and P2^neo/neo ^= 1.51 ± 0.31; P < 0.001). To examine P1/P2 usage in the DN sub-groups we used flow cytometry to sort E15.5 thymocytes based on surface expression of CD25 and CD44 (as shown in Fig. 5A), and levels of P1 and P2 transcripts was determined. The levels of P1 and P2 expression did not differ significantly between DN3 and DN4 subgroups and was similar to the level shown in Fig. 1D.

Next we examined the CD8/CD4 double positive population. At E16.5, P2^neo/neo ^embryos displayed a 2.5-fold reduction in the proportion of double positive (DP) CD4^+^/CD8^+ ^thymocytes as compared to WT (Fig. 5B). At E17.5 the decrease in proportion of DP thymocytes in P2^neo/neo ^embryos was more pronounced and accompanied by a marked increase in an abnormal population of CD4^+^/CD8^low ^cells (Fig. 5B). A similar subpopulation of immature CD4^+^/CD8^low ^was previously observed in mice with thymocyte specific targeting of Runx1 [13,28] and in mice bearing a hypomorphic *Cbfβ *allele [49]. It was postulated that this abnormal CD4^+^/CD8^low ^population represents immature DP thymocytes in which CD8 expression was diminished and *CD4 *expression was partially de-repressed [13]. Delayed differentiation of P2^neo/neo ^thymocytes was still apparent at post-natal day (P) 1.5 (Fig. 5B), but recovery to WT proportions was noted at P3.5.

Taken together, the change in the proportion of DN3/DN4 thymocytes at early thymopoiesis, the decrease in DP CD4/CD8 thymocytes and the increase in the abnormal SP CD4^+^/CD8^low ^population, indicate that during embryonal thymopoiesis P2 activity is also required for regulation of CD4/CD8 expression and thereby for the proper differentiation of DN and DP thymocytes.

### Reduction in committed T-cell precursors (TCPs) in P2^neo/neo ^fetuses

**Figure 6:**
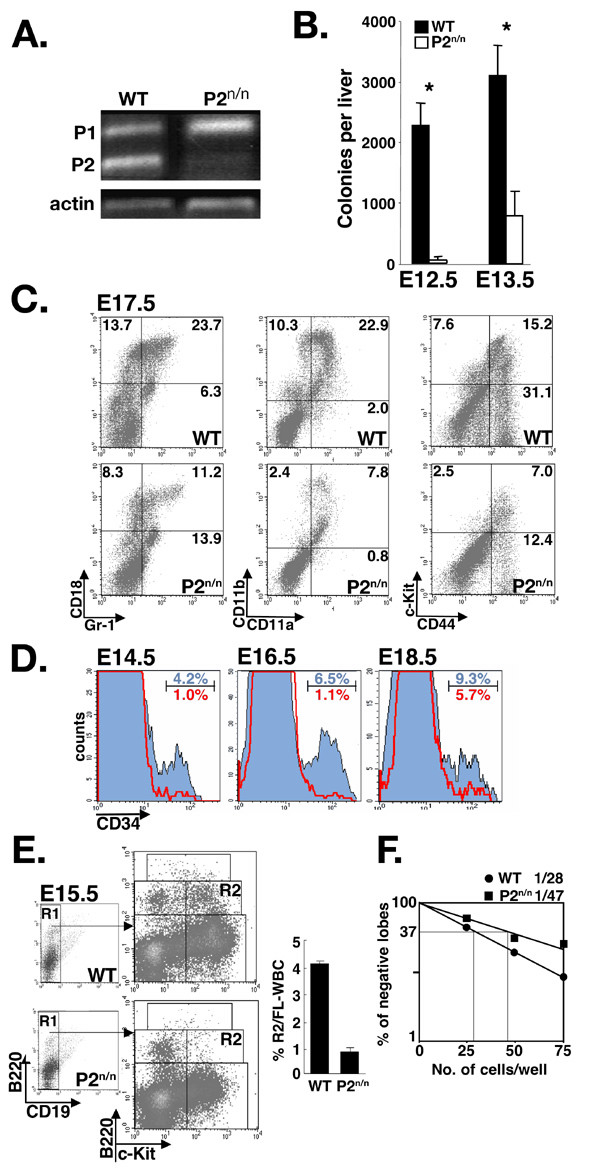
**P2-mediated embryonal expression of *Runx1 *is required for development of FL HPC**. (A) RT-PCR analysis of RNA from E13.5 FL cells of WT and P2^neo/neo ^embryos. P2-mediated transcription in P2^neo/neo ^was largely attenuated, whereas P1-mediated transcription was apparently unaffected. (B) Colony forming activity of FL hematopoietic stem/progenitor cells. Colonies (>30 cells in size) were scored on day 7 of incubation. Shown are average numbers of colonies per FL ± S.E. of at least three different WT or P2^neo/neo ^embryos at E12.5 and E13.5. The difference between WT and P2^neo/neo ^colony number is significant (*) at P < 0.001 by Student's t test. (C and D) Altered expression of surface antigens on P2^neo/neo ^FL HPC. (C) Expression profiles of Gr-1, CD18, CD11b, CD11a, CD44 and c-Kit, in FL HPC from E17.5 WT and P2^neo/neo ^embryos. Representative data of at least three different experiments are shown as FACS dot plots; percentages of cells in each quadrant are indicated. Note the 2-fold decrease in the proportions of Gr-1^+^/CD18^+ ^and CD11a^+^/CD11b^+ ^(left panels) as well as CD44^+^/c-Kit^+ ^(right panel), FL cells in P2^neo/neo ^compared to WT. (D) Proportion of CD34^+ ^FL HPC. Profiles of WT and P2^neo/neo ^FL HPC, filled (blue) and unfilled (red) histograms, respectively, at E14.5, E16.5 and E18.5. Shown are representative results of at least three different experiments. Numbers at the upper right corners are percentages of CD34^+ ^cells in WT (upper) and P2^neo/neo ^(lower). Note the marked decrease in the proportions of P2^neo/neo ^CD34^+ ^HPC. (E) Analysis of committed FL T-cell precursors (TCPs). FACS analysis showing the distribution of B220^+^/c-Kit^+^/CD19^- ^FL HPC of E15.5 WT and P2^neo/neo ^embryos. Gated CD19^- ^precursors (R1) were analyzed for c-Kit and B220 expression. Note the 4-fold decrease in the proportions of c-Kit^+^/B220^low^/D19^- ^TCPs (R2) in P2^neo/neo ^FL HPC. Data shown on the right are average ± S.E. of four independent experiments using four different mice. The difference between the percentages of TCPs from WT and P2^neo/neo ^FL HPC is significant at P < 0.005 by Student's t test. (F) Reduced capacity of P2^neo/neo ^FL HPC to colonize FTOC. Isolated E15.5 FL HPC from WT or P2^neo/neo ^embryos were set to differentiate, under limiting dilution conditions, for 16 days in E14.5 WT FTOC. Shown are percentage of unsuccessfully populated lobes per cultured cells and the calculated frequencies of three different independent experiments.

Runx1 is essential for embryonal hematopoiesis and Runx1^-/- ^mice lack definitive hematopoiesis as well as hemapoietic colony forming activity *in vitro *[16,17,19,20]. In developing FL, Runx1 is expressed in HSC and in early progenitors [9,16,17], and in WT FL both promoters are active (Fig. 6A and [50]. In hematopoietic FL cells of P2^neo/neo ^embryos, P1-transcription was not affected, as was noted earlier for P2^neo/neo ^thymocytes (Fig. 1G), whereas P2 activity was largely attenuated (Fig. 6A). To assess hematopoietic activity, individual FLs from WT and P2^neo/neo ^embryos were isolated and analyzed for *in vitro *colony formation. As shown in Fig. 6B, the number of colonies grown in P2^neo/neo ^cultures was significantly lower compared to WT. However, because the size of the colonies in P2^neo/neo ^and WT cultures was similar (data not shown), the results indicate that P2^neo/neo ^FL cells contained fewer progenitors compared to WT. This conclusion was supported by the finding that E17.5 and E18.5 P2^neo/neo ^embryos showed marked reduction (~50 ± 3%) in the proportion of Gr-1^+^/CD18^+ ^and CD11a^+^/CD11b^+ ^cells compared to WT littermates (Fig. 6C and data not shown).

We next assessed the effect of the P2^neo/neo ^hypomorph on the development of FL HPC. Runx1 expressing FL HPC also express CD34, c-Kit and CD44 [16,17]. In E17.5 and E18.5 P2^neo/neo ^embryos, a 2-fold reduction in the proportion of CD44^+^/c-Kit^+ ^HPC compared to WT littermates was observed (Fig. 6C), and even greater reduction was observed in CD34 positive cells at E14.5–E18.5 (Fig. 6D and data not shown). FL HPC that are B220^low^/c-kit^+^/CD19^- ^represent an HPC population restricted to the T-cell and natural killer cell lineages [51]. These FL progenitors, termed T-cell precursors (TCPs), are thought to represent the immediate developmental step before thymic immigration. Because the hypocellularity of P2^neo/neo ^thymus was noted at the earliest developmental stages (Figs. 1H and 2B), the proportion of TCPs in WT and P2^neo/neo ^was compared. FACS analysis of B220^low^/c-kit^+^/CD19^- ^E15.5 FL cells, revealed a fourfold reduction in the proportion of P2^neo/neo ^TCPs compared to WT (Fig. 6E). These results indicated that the proportion of Runx1 expressing FL HPC, particularly the TCP subset, was markedly reduced in P2^neo/neo ^compared to WT. This conclusion was supported by *in vitro *FTOC progenitor assay, which monitors the capacity of FL TCPs to reconstitute thymic lobes [51-54] (Fig. 6F). When E15.5 WT and P2^neo/neo ^FL cells were cultured under limiting dilution conditions in FTOCs, the frequency of successfully populated thymic lobes was lower for P2^neo/neo ^TCPs as compared to WT (~1 in 47 P2^neo/neo ^cells vs. 1 in 28 WT cells) (Fig. 6F).

Additionally, when day 16 FTOCs cells were obtained and CD4/CD8 positive cells analyzed by FACS, an abnormal differentiation of P2^neo/neo ^thymocytes was observed with increased proportion of the abnormal population DP CD4^+^/CD8^low ^and a twofold reduction in the proportion of SP CD4^+ ^or CD8^+ ^compared to WT (not shown). This FTOC result corresponds well with the *in vivo *delayed maturation of P2^neo/neo ^fetal thymocytes described in Fig. 5. Collectively, these data indicate that in P2^neo/neo ^embryos the development of FL HPC of different lineages was impaired.

### Runx1 P2^neo ^allele failed to rescue the embryonal lethality phenotype of Runx1^-/- ^mice

**Figure 7:**
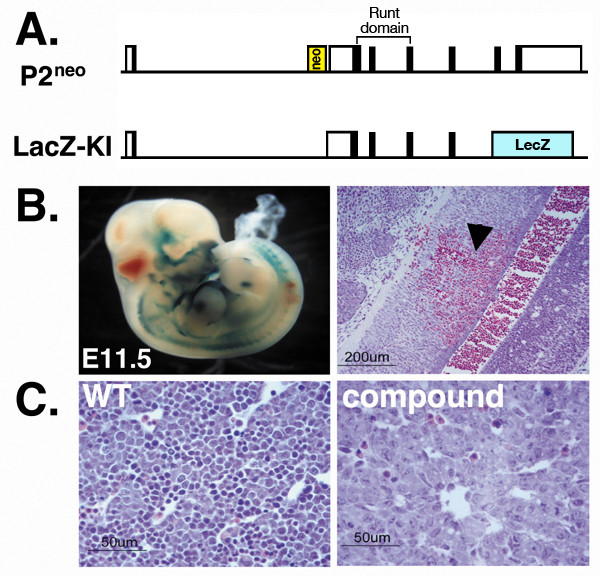
***Runx1 *P2^neo ^allele failed to rescue the embryonal lethality phenotype of *Runx1*^-/- ^mice**. (A) Schematic representation of *Runx1 *locus in the two *Runx1 *mutant strains used to generate the compound mutant strain *Runx1*^lz^;*Runx1*^P2neo^. (B) *Runx1*^lz^;*Runx1*^P2neo ^die at E11.5 to E12.5 due to hemorrhages in the CNS and/or lack of FL hematopoiesis. Left: Lateral view of a whole mount compound *Runx1*^lz^;*Runx1*^P2neo ^E11.5 embryo stained for β-galactosidase activity. Hemorrhages are seen in the 4^th ^ventricle, the ventral metencephalon and spinal cord. Right: Hematoxylin and eosin (H&E) staining of a section through the spinal cord exhibits focal hemorrhage (arrow head). (C) H&E staining of WT (left view) and *Runx1*^lz^;*Runx1*^P2neo ^(right view) E12.5 fetal liver. Note the absence of definitive hematopoietic precursors (deep purple cells) in *Runx1*^lz^;*Runx1*^P2neo ^FL as compared to WT. The data demonstrate that activity of a single *Runx1 *P1 was not sufficient to rescue the embryonal lethal phenotype of Runx1^-/- ^mice.

Homozygous disruption of *Runx1 *results in a complete absence of FL hematopoiesis and the mice die between E11.5 and E12.5 of hemorrhages in the central nervous system [19,20]. P2^neo/neo ^mice were born and showed no sign of hemorrhages, indicating that expression levels of *Runx1 *in homozygous P2^neo/neo ^during early embryogenesis were sufficient to rescue the Runx1^-/- ^phenotype. This occurrence raised the question of whether Runx1 levels provided by activity of a single P1 will also be sufficient to rescue the null phenotype. To address this issue we generated a compound mutant strain *Runx1*^lz^; *Runx1*^P2neo ^by mating *Runx1*^lz/+ ^mice [16] with Runx1^P2neo/+^. These mice had one null allele expressing a fused Runx1-LacZ protein and one P2^neo ^allele expressing WT P1 (Fig. 7A). Compound mutant *Runx1*^lz^;*Runx1*^P2neo ^embryos died between E11.5 and E12.5 and suffered from hemorrhages in the nervous system (Fig. 7B). The most extensive bleedings was seen in the 4^th ^ventricle, the ventral metencephalon and spinal cord. Moreover, the liver of E12.5 compound mutant embryos was depleted of hematopoietic cells (Fig. 7C), indicating lack of definitive hematopoiesis, similar to the previously reported *Runx1*^-/- ^phenotype [16,19,20]. These results indicated, that during midgestation, angiogenesis and definitive hematopoiesis are highly sensitive to the dosage of Runx1 as activity of a single P1 was not sufficient to rescue the *Runx1*^-/- ^phenotype. This conclusion is consistent with the previously reported data which was based on transplantation assays [17,18,55]. This observation may also pertain to the unique role of P2 activity during midgestation angiogenesis and/or early hematopoiesis.

### Early lethality phenotype of P2^neo/neo ^neonates is rescued by excision of the neo cassette in early T cell progenitors

**Figure 8:**
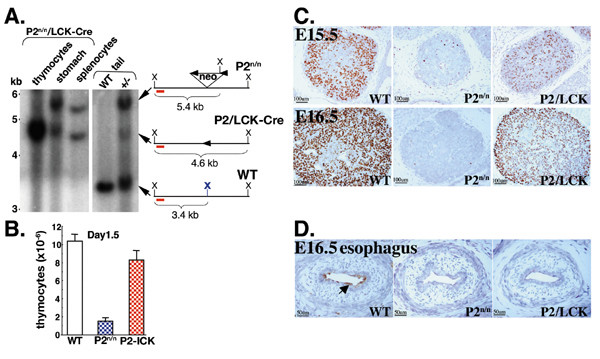
**Rescue of P2^neo/neo ^dependent thymic defect by T-cell specific removal of the *neo *cassette**. (A) Specific excision of the *neo *cassette in P2/Lck-Cre mice. Southern blot analysis using a probe spanning the region indicated by the red bar assessed the level of *neo *excision in thymocytes, stomach epithelium and splenocytes of 4 week-old P2/Lck-Cre mice. The positions of *Xba*I cleavage sites are shown (X). The probe hybridized to a 3.4 Kb- and a 5.4 Kb *Xba*I genomic fragments derived from WT and P2^neo/neo ^allele, respectively. Insertion/excision of the *neo *cassette by Lck-Cre eliminated the middle *Xba*I site (shown in WT as bold blue) generating the 4.6 Kb fragment derived from the P2/Lck-Cre allele. (B) Regeneration of thymus cellularity in P2/Lck-Cre mice. At day-1.5 the number of thymocytes in P2/Lck-Cre thymic lobes was near normal compared to WT littermates. Data shown are average ± S.E. of five independent experiments using five mice of each genotype (WT, P2^neo/neo ^and P2/Lck-Cre). The difference between WT and P2^neo/neo ^is significant at P < 0.001 by Student's t test. (C) Recovery of thymopoiesis and thymic organogenesis in P2/Lck-Cre embryos. Histological analysis and *Runx1 *expression in thymic lobes derived from E15.5 and E16.5 WT, P2^neo/neo ^and P2/Lck-Cre embryos. Shown are H&E stained transverse sections immunostained for Runx1. (D) Runx1 expression was not rescued in P2/Lck-Cre epithelial cells. IHC analysis of Runx1 expression in esophagi derived from E16.5 WT, P2^neo/neo ^and P2/Lck-Cre embryos. Transverse sections showing Runx1 expression in epithelial cells lining the lumen of WT (arrow), but not of P2^neo/neo ^or P2/Lck-Cre esophagus. Several other tissues including stomach and nasal cavity were similarly analyzed and revealed no expression of Runx1 in epithelia of P2^neo/neo ^or P2/Lck-Cre mice (not shown).

As noted earlier, P2^neo/neo ^neonates die within a few days after birth. The reasons for this early lethality are not clear. While P2-mediated expression diminished in several P2^neo/neo ^tissues, including stomach, intestine and kidney (Fig. 1), the most striking abnormalities were noted in thymic development and thymopoiesis. We thus addressed whether these abnormalities could be rescued by specific removal of the *neo *cassette in early thymopoiesis and whether this occurrence will extend the life span of P2^neo/neo ^neonates. *Runx1-*P2^neo/neo ^mice were mated with a *Lck-cre *transgenic line [56] to excise the *neo *and regain P2 activity in thymocytes (Fig. 8A). Contrary to P2^neo/neo ^mice, the P2/*Lck-Cre *mice developed normally and outwardly were indistinguishable from WT littermates. Nevertheless, embryonal thymopoiesis of P2/*Lck-Cre *was not completely normal and embryos exhibited a lower number of Runx1 expressing thymocytes (Figs. 8B and 8C). This may be due to an incomplete excision of the *neo *cassette in all T cell progenitors. Significantly, however, Runx1 expression level in P2/*Lck-Cre *was sufficient to allow for normal thymic development. Of note, P2-mediated Runx1 expression in other Runx1 expressing cells, such as epithelial cells, was not rescued in P2/*Lck-Cre *mice (Fig. 8D and data not shown).

## Discussion

Runx1 is an important cell-linage specific regulator of definitive hematopoiesis and thymopoiesis. The unique spatio/temporal expression pattern of Runx1 is attained through alternative usage of its two promoters, P1 and P2 [29,30,57] that also impacts on translation efficacy and repertoire of protein isoforms [30,57]. While it was clear that transcriptional regulation of Runx1 expression is an important element of its biological function, little was known about the role of the two promoters during development, and about how and when they are differentially regulated. Is the alternative promoter usage of human and mouse *RUNX1*/*Runx1 *a reflection of vertebrates' higher complexity? Differential usage of alternative promoters enables diversified transcriptional regulation within a single locus and may thus serve as a molecular basis for the higher complexity of certain vertebrates' systems, such as the immune system. Indeed, while the *RUNX *genes are highly conserved throughout the animal kingdom, more primitive animals such as the nematode *C. elegance *and the sea urchin contain only one gene regulated by a single promoter [3,8]. This single promoter is the equivalent of vertebrates' P2.

### Differential P1/P2 usage during embryogenesis

The use of hypomorphic alleles for studying essential genes at the organismal level is a well-established approach. We employed mice homozygous for a hypomorphic *Runx1 *allele, which caused a profound attenuation of P2 activity, to investigate the biological role of P2 during embryogenesis. Specifically, we analyzed the impact of the resulting hypomorphic Runx1 expression on FL HPC development and early thymopoiesis. We found that during embryogenesis *Runx1 *is developmentally regulated through alternative usage of P1 and P2. In certain tissues, including kidney, tongue and liver both P1 and P2 are active, whereas in others such as intestinal epithelium and early thymocytes P2 activity predominates. Contrary to Runx1 null mice, in which embryonic lethality occurs at ~E12.5 [19,20], P2^neo/neo ^mice develop to term, implying that during early development the activity of P1 prevails. This conclusion is supported by the finding that both P1- and P2-transcripts are found in FL of E12.5 embryos [50]. The notion that both P1 and P2 are active during early hematopoiesis correlates well with the finding that P2^neo/neo ^embryos displayed moderate and severe hematopoietic defects in the liver and thymus, respectively.

### P2 activity is essential for embryonal thymopoiesis and thymus development

Thymic development in the mouse begins at ~E9.5 [42]. Runx1 is highly expressed in thymocytes, but not in thymus epithelium [9,13,14]. Using *in-situ *hybridization, RT-PCR and IHC we demonstrate that during early thymopoiesis activity of P2 predominates. The P1 is upregulated at E17.5 and its activity persists thereafter, while the activity of P2 declines after birth. At E11 the thymic rudiment is colonized by the first wave of committed T-cell precursors (TCP) [54] which endure in the thymus until E17 [58]. It is tempting to speculate that P2 activity is confined to descendants of the first wave TCP, whereas P1 is active in thymocytes originating in the second wave. Consistent with this hypothesis, the first wave P2^neo/neo ^thymocytes contained a markedly reduced level of Runx1 compared to WT, whereas in second wave thymocytes the level was similar to WT due to switch-on of P1 at E17.5. These second wave thymocytes grow exponentially after birth, when P1 is more active, and become the major cell population in the thymus.

During early stages of thymus development (i.e. E14.4 to E16.5) a marked reduction in thymic cellularity was observed in P2^neo/neo ^embryos. We show that this occurrence was due to enhanced apoptosis of P2^neo/neo ^thymocytes. But why were E14.4 to E16.5 P2^neo/neo ^thymocytes more susceptible to apoptosis than WT cells?

Selection processes in the thymus ensure that peripheral T cells are responsive to foreign antigens but tolerant to self-antigens. This occurs through positive and negative β-selections. Thymocytes, whose TCR engaged peptide/MHC molecules productively, escape their inherent programmed cell death and subsequently are induced to proliferate. On the other hand, thymocytes that react strongly with self-peptide-MHC molecules are actively eliminated [59-61]. According to the avidity model, selection occurs only when signals received through the TCR fall between two thresholds: signals below the one are incompatible with survival (death by neglect), while signals above the other result in deletion [47,62,63]. We hypothesize that the elevated TCR levels in P2^neo/neo ^thymocytes disturbed this delicate balance and disposed a larger proportion of P2^neo/neo ^T cells to apoptosis.

Several studies in transfected cells have demonstrated the involvement of Runx1 in transcription regulation of TCRs expression [8]. Specifically, we have shown that Runx1 can act as a transcriptional repressor in TCR regulation [4]. Thus, it is conceivable that the increased levels of TCRs in embryonic P2^neo/neo ^thymocytes resulted from the reduced Runx1 expression in these cells, and that the higher TCRs levels lead to a higher avidity of TCR-MHC interactions, which programmed the cells to apoptosis. This hypothesis is supported by *Runx1*^F/F^/Lck-Cre mice, which show enhanced negative selection of Runx1^-/- ^thymocyte [13], and by the finding that upon recovery of Runx1 expression in P2^neo/neo ^thymocytes, TCR expression and apoptosis resumed WT levels. Potentially related, mice homozygous for a knock-in *Runx1 *cDNA that are lacking the C-terminal VWRPY, exhibit thymic hypocellularity [28]. The VWRPY motif, which is highly conserved among all RUNX proteins, is essential for recruitment of the transcriptional co-repressor Gro/TLE [4,7,64] and subsequent repression of target genes [4,7]. Thymic hypocellularity was also noted in mice bearing a hypomorphic allele of *Cbfβ*, the non-DNA binding partner of Runx1, which is essential for the function of RUNX proteins [49].

In addition to the marked hypocellularity, thymi of P2^neo/neo ^embryos contained an increased proportion of DN3 (CD25^+^/CD44^-^) thymocytes, a reduced proportion of DP CD4^+^/CD8^+ ^thymocytes and a pronounced increase in an abnormal population of immature CD4^+^/CD8^low ^cells. The increase in DN3 and CD4^+^/CD8^low ^thymocyte populations was previously observed in adult *Runx1*^F/F^/Lck-Cre mice due to diminished Runx1 in DN thymocytes and the consequent derepression of *CD4 *[13]. More recently a similar increase in CD4^+^/CD8^low ^thymocytes was also observed in fetuses of *Cbfβ*^rss/- ^mice bearing the hypomorphic *Cbfβ *allele [49], indicating that Cbfβ is required for *CD4 *silencing by Runx1. Abnormally high proportion of CD4^+ ^thymocytes was also noted in embryos of the Runx1 knock-in mutant lacking the VWRPY motif [28]. This finding suggests that Runx1-mediated repression of *CD4 *in DN thymocytes involves the recruitment of Gro/TLE, as in case of Runx3-mediated silencing of *CD4 *in DP thymocytes [7].

### Attenuation of P2 activity affects development of FL HPC

Runx1 is essential for the emergence of embryonal HSC [16-18]. Analysis of FL hematopoiesis in P2^neo/neo ^embryos revealed that the emergence and commitment of HPC to either myeloid or lymphoid lineages was not affected by attenuation of P2. However, the noted delay in differentiation and expansion of various P2^neo/neo ^progenitor populations indicated that development of FL HPC was sensitive to diminution in P2 activity. Interestingly, the overall phenotype of FL HPC in P2^neo/neo ^embryos resembled the phenotype observed by Cai et al [18] in heterozygous Runx1^+/- ^fetuses, suggesting a gene dosage effect. In developing FL activity of P1 emerged early and persisted throughout gestation [50]. As P1 activity was largely unaffected in P2^neo/neo ^HPC, it is conceivable that the hypomorphic expression in P2^neo/neo ^FL creates conditions resembling haploinsufficiency [18], in line with the large body of literature documenting hematopoietic defects due to hemizygous dosage of Runx1 [2,17,20,65]. However, it is worth noting that the effect of P2^neo/neo ^hypomorph is not merely through gene dosage, because contrary to Runx1^+/- ^the P2^neo/neo ^neonates die within a few days after birth.

### Rescue of the early lethality of P2^neo/neo ^neonates

Despite the diminished P2-activity and reduced Runx1 dosage, P2^neo/neo ^embryos developed to term. Thus the residual P2 activity plus the apparent WT expression of P1 were sufficient to support normal development of P2^neo/neo ^embryos through gestation. However, inactivation in P2^neo/neo ^embryos of one P1 allele, as in the compound mutant *Runx1*^lz^*;Runx1*^P2neo^, which further reduced Runx1 dosage, caused hemorrhages in the nervous system and mid-gestation (E11.5–E12.5) lethality. This result defined the threshold level requirement of Runx1 for rescue of the nervous system hemorrhages. While P2^neo/neo ^embryos developed to term, neonates die within a few days after birth. Specific removal of the floxed P2-*neo *cassette by crossing P2^neo/+ ^mice with *Lck-Cre *transgenics resumed P2 expression in early thymopoiesis, allowed for normal thymic development and rescued the neonatal lethality. Of note, the neonatal lethality was rescued even though expression of P2 in the gastrointestinal tract of P2^neo/neo^/Lck-Cre mice was not resumed. This occurrence indicated that lethality of P2^neo/neo ^neonates was not due to lack of Runx1 expression in gastrointestinal tract epithelium, despite the striking reduction of nutrients in the gastrointestinal tract of P2^neo/neo ^neonates (see Additional File 2C and 2E). As athymic nude mice live through adulthood, it is conceivable that impaired thymopoiesis in P2^neo/neo ^embryos, especially at the negative selection step, resulted in emergence of pathogenic T cells that cause the neonatal lethality. Finally, as the N-terminus of Runx1 proteins encoded by P2-mRNA differs from that of P1 [3], it is tempting to speculate that the hematopoietic phenotypes of P2^neo/neo ^embryos were caused not only by gene dosage, but are due to the lack of P2 isoforms. Elucidating the specific role of the P1- and P2-protein isoforms in Runx1 biology, particularly early thymopoiesis, awaits further investigations.

## Conclusion

Differential usage of alternative promoters enables diversification of transcriptional regulation within a single locus and thereby plays a significant role in the control of gene expression. During mouse embryogenesis the spatio/temporal expression of *Runx1 *is developmentally regulated by alternative usage of two mutually distinct promoters, P1 and P2. These promoters are not only differentially regulated, but also give rise to mRNAs with variant 5'-UTRs and to structurally different protein isoforms. Our studies support a model whereby attenuation of P2 affects mRNA levels and protein isoforms repertoire within the cell and thereby creates the hematopoietic phenotype of P2^neo/neo ^embryos. The findings provide *in vivo *evidence for the non-redundant function of P1 and P2 and underscore the significance of alternative promoter usage in *Runx1 *biology.

## Methods

### Generation of Runx1 P2^neo/neo ^mice

Runx1 genomic clone was isolated from 129/Sv-derived ES cells DNA and a 7.2 Kb *EcoRI-PstI *fragment spanning 3 Kb upstream of P2, exon 2 and 2.4 Kb of intron 2 [57] was subcloned into pBluescript. A 2 Kb *pgk-neo *cassette was inserted into the XbaI site at nucleotide 27675861 of the mouse genome (accession # NT-039625). R1 ES cells were transfected with the targeting construct and homologous recombinants were evaluated by Southern blot analysis. Targeted ES cells were used to create several chimeras that passed the mutated Runx1 allele to their progeny. Two independent P2^neo/neo ^mouse lines from two different ES clones were established and bred onto ICR and MF1 strains. As identical phenotypes were observed with mutant mice generated from either of the two-targeted ES cell lines, and with mutant mice bred on either ICR or MF1 background, in most cases we have combined the data. Genotypes were examined by PCR using genomic tail DNA and when applicable verified by Southern analysis. The noon of vaginal plug was considered E0.5. To remove the *neo *cassette, homozygotes P2^neo/neo ^mice were crossed onto the transgenic Cre-deleter mouse strain PGK-Cre [40] generating mice lacking the *neo *cassette. Cre-mediated excision of *neo *was confirmed by PCR. Mice were bred and maintained in a pathogen-free facility. All experiments involving mice were approved by the Institutional Animal Care and Use Committee (IACUC) of the Weizmann Institute.

### Isolation of hematopoietic cells

Thymic lobes and livers of embryos or newborns were aseptically removed, and a single-cell suspension was generated. When required, red blood cells were lyzed with 1:9 volumes of PBS:RCLB (0.9% NH_4_Cl, 0.1% KHCO_3_, 0.0037% EDTA, pH7.2).

### Protein and RNA analysis

Tissues were collected, washed once in PBS, and proteins or RNA were extracted. Protein extracts were subjected to Western blot analysis as described previously [30,35]. Poly(A)^+ ^RNA was purified from 150 mg of total RNA using oligo(DT) magnetic beads (Dynal. Oslo, Norway) and subjected to Northern blot analysis as previously described [30,31]. WT thymocytes expressed four alternatively spliced isoforms of *Runx1 *transcripts, of which the 2 Kb- and 6 Kb result from P1-mediated transcription, while the 4 Kb- and 8 Kb from P2 activity [31].

### RNA preparation and RT-PCR analysis

Total RNA isolated using Tri-Reagent™ (MRC) was reverse transcribed using SuperScript™ II reverse transcriptase (Invitrogen) and resulting cDNA was subjected to PCR reactions using the SuperTherm polymerase (Hoffman-La-Roche). To amplify *Runx1 *specific sequences, we used a common antisense primer that hybridizes to a sequence in the Runt domain exon 4 with two different sense primers that were complementary to either P1- or P2 5'UTR. Thereby the expression of both P1 and P2 regions were simultaneously monitored, providing a reliable readout of their relative expression levels. The sequences of primers and conditions used for PCR analysis of *Runx1 *and *TCR *are as follows: Primers for *Runx1 *amplification: 5'-GAAACGATGGCTTCAGACAGC-3' (P1-sense), 5'-CTTGGGTGTGAGGCCGATCC-3' (P2-sense), and 5'-ATGACGGTGACCAGAGTGCC-3' (runt domain antisense). β-actin was used as an internal reference, with primers: 5'-GATGACGATATCGCTGCGCTG-3' (sense) and 5'-GTACGACCAGAGGCATACAGG-3' (antisense). The PCR conditions used were as follow: 94°C for 3 min, followed by 40 cycles of 94°C for 45 s, 57°C for 45 s and 72°C for 45 s. Primers for *TCRs *amplification: 5'-TCCTCCCCCAACAGGTAGCT-3' (Pre TCRα-sence), 5'-GGCAAACCACCAGCATGTG-3' (Pre TCRα-antisence), 5'-TCTCATAGAGGATGGTGGTGGCAGACA-3' (constant TCRb-sence), 5'-CTGCTACCTTCTGGCACAATCC-3' (constant TCRβ-antisence), 5'-AGGCTTGATGCAGACATTTCCCCC-3' (constant TCRγ-sence) and 5'-CTTGCCAGCAAGTTGTAGGCTTGG-3' (constant TCRγ-antisence). The PCR conditions were as follow: 94°C for 3 min, followed by increasing number of cycles (25, 30, 35) of 94°C for 45 s, annealing for 45 s and 72°C for 60 s. Annealing temperatures were the following: 56°C-TCRα, 55°C-TCRβ and 59°C-TCRγ. All PCR products were analyzed by 1.5% agarose gel and visualized by staining with ethidium bromide.

### In-Situ hybridization

*In situ *hybridization with Digoxigenin (DIG)-labeled antisense and sense Riboprobes made of the P2-5'UTR, spanning nucleotide No. 27,373,200-27,374,550 in Genbank Accession # Mm16_39665_36 (see S1A), was performed according to published procedures [66], using 14 μ frozen sections. DIG labeled Riboprobes were detected by anti-DIG antibody conjugated to alkaline phosphatase (Roche), and developed with nitro blue tetrazolium/5-bromo-4-chloro-3-indole-phosphate.

### Histology and immunohistochemistry

Histology and immunohistochemistry were performed as previously described [9,10]. Polyclonal anti Runx1 antibodies (1:1000) [9,35], and anti Ki-67 antibodies (1:50) (DakoCytomaion), were incubated overnight at room temperature in blocking solution of PBS containing 0.5% Triton X-100 and 3% heat inactivated normal goat serum for anti Runx1, or normal rabbit serum for anti Ki-67 [67]. Bound antibody was detected using biotinylated goat anti-rabbit IgG secondary antibodies for Runx1, and goat anti-rat IgG for Ki-67. The ABC complex from Vectastain kit (Vector Laboratories, Burlingame, CA) was used for peroxidase detection. Slides were counterstained with hematoxylin.

### Flow cytometric analysis

Flow cytometry was performed as previously described [14,68], using the following mAbs from Pharmingen (San Diego, CA): CD4-FITC, CD8-PE, CD44-FITC, CD25-PE, TCRβ-FITC, TCRβ-biotinylated, TCRγδ-PE, c-KIT-PE, CD19-FITC, B220-biotinylated, Gr-1-FITC, CD18-PE, CD11a(LFA-1)-FITC, CD11b(Mac-1)-PE, CD34-FITC, streptavidin-APC.

### Proliferation assay and apoptosis analysis

Proliferation assay was conducted as before [14]. 10^3 ^FACS sorted Annexin V negative cells were assayed for proliferation using [^3^H]thymidine (specific activity 25 Ci/mmole). Apoptosis was monitored by double staining with Annexin V and PI. Annexin V-FITC-conjugated anticoagulant (Annexin-V-Fluos Roche, Mannhein, Germany) was used according to manufacturer's instructions. *In situ *apoptotic cells were detected by staining paraffin sections of thymic lobes with ApopTag Apoptosis Detection System (Biotech). Activated caspase-3, a key apoptotic marker, was detected by IHC of paraffin sections with polyclonal antibodies against activated caspase-3 (Cell Signaling Technology).

### Fetal thymic Organ Culture (FTOC)

Isolation of WT E14.5 thymic lobes, treatment with 1.35 mM 2'-deoxyguanosine (2-dG) (Sigma, St Louis, MO) and incubation of lobes with WT or P2^neo/neo ^FL HPC by means of the hanging drop procedure, were performed as previously described [69]. After 16 days in organ culture, the lobes were removed, rinsed in medium and analyzed for newly populated thymocytes. Limiting dilution assay was performed as described by Jenkinson et al [52] and Dougai et al [51,54]. The frequency of TCPs per thymic lobe was deduced based on the Poisson probability distribution as previously described [51,54].

### Colony forming unit (CFU) assay

10^5 ^FL cells were cultured in 1 ml of single layer DMEM culture medium containing 0.33% agar and 20% horse serum supplemented with 50 ng/ml stem cell factor, 20 ng/ml IL-3, 25 ng/ml IL-6, 5 ng/ml GM-CSF, and 2 ng/ml G-CSF to provide optimal conditions for multiplication and differentiation of multipotential hematopoietic progenitors of definitive hematopoiesis [19]. The number of colonies was determined after 7 days of incubation.

## Authors' contributions

AP created the mutant mice, carried out the phenotypic analysis and drafted the manuscript. JL participated in hematopoietic progenitor differentiation analysis, assisted in data analysis and in drafting the manuscript. CX performed the in situ hybridization experiments. DG carried out protein analysis. OB analyzed the histology and immunohistochemistry data and conceived the pertained parts in the manuscript. VN and DL participated in molecular genetic studies and YG conceived and supervised the study, evaluated the data and wrote the manuscript. All the authors read and approved the final manuscript.

## Supplementary Material

Additional file 1 ***Insertion of a neo cassette into Runx1 P2 region inhibits transcriptional activity in transfected cells***. Two reporter plasmids were constructed (designated P2-Ren and P2^neo^-Ren) in which a Runx1 genomic region spanning 3 Kb upstream of P2 transcription start sites regulated the expression of Renilla luciferase gene. (A) Schematic drawing of P2-Ren and P2^neo^-Ren reporter plasmids. Both contained a genomic region which spans 3-kb upstream of P2 transcriptional start site (TSS), complete exon 2 and 2.4-kb of intron 2 [57]. The data show that in epithelial (HEK 293) and osteoblast (HOS) cell lines the inserted neo cassette had no effect on P2 activity whereas in T-cell (Jurkat), myeloid (U937) and neuronal (PC12) cell lines the neo cassette significantly attenuated the activity of Runx1 P2. Click here for file

Additional file 2 **Generation of *Runx1 *P2^neo/neo ^mice and phenotypic analysis of mutant newborns**. (A, B) Scheme of genomic organization of *Runx1 *outlining the steps employed to generate the mutant P2^neo ^locus and Southern blot analysis of genomic DNA identifying homologous recombination in ES clones and in newborn mutant mice. The region within P2-5'UTR (accession # D26532), which was used as a probe for *in-situ *hybridization is indicated on the targeting construct (striped bars), whereas the primers used for RT-PCR analysis are indicated on the genomic scheme (arrow heads). F1 heterozygotes *Runx1*^P2neo ^(P2^neo^) were intercrossed and all three genotypes were detected in F2 litters. Transmission of the mutant allele roughly followed a Mendelian inheritance pattern, indicating that mice homozygous for the *Runx1 *mutant allele were born. The *neo *gene was excised (Mutant locus->Neo minus locus) by crossing heterozygous *Runx1 *P2^neo/+ ^mice onto the appropriate *Cre *transgenic mice as described in results. (C) Early neonatal lethality of homozygous *Runx1 *P2^neo/neo ^mice. P2^neo/neo ^neonates exhibit marked growth retardation and die within few days after birth. At birth mutant mice were as active as their littermates, exhibited suckling behavior and had milk in their stomachs. However, at day 2 the amount of milk in the stomach of P2^neo/neo ^mice drastically decreased. (D) Body weights of newborn WT and P2^neo/neo ^mice during the first three days as observed in two litters (n = 14). Newborns were weighed at the indicated time after birth. WT and P2^neo/+ ^mice gained weight, whereas P2^neo/neo ^did not. (E) Stomach and duodenum of P2.5 WT and P2^neo/neo ^littermate mice. Volume of milk in P2^neo/neo ^stomach was significantly lower compared to WT. To further characterize the phenotype/genotype relationships in P2^neo/neo ^mice, the *neo *gene was removed, as described in the results and shown in (B). As removal of the *neo *gene rescued the early lethality phenotype of P2^neo/neo ^newborns, we concluded that the P2^neo/neo ^phenotype resulted from the presence of *neo *in the P2 region. Click here for file
