# Supplementary material for: Developmentally regulated promoter-switch transcriptionally controls Runx1 function during embryonic hematopoiesis
Source: BMC Dev Biol. 2007 Jul 12;7:84. doi: 10.1186/1471-213X-7-84 (PMC1941738; doi:10.1186/1471-213X-7-84)
Supplement: Additional file 1 — Insertion of a neo cassette into Runx1 P2 region inhibits transcriptional activity in transfected cells. Two reporter plasmids were constructed (designated P2-Ren and P2neo-Ren) in which a Runx1 genomic region spanning 3 Kb upstream of P2 transcription start sites regulated the expression of Renilla luciferase gene. (A) Schematic drawing of P2-Ren and P2neo-Ren reporter plasmids. Both contained a genomic region which spans 3-kb upstream of P2 transcriptional start site (TSS), complete exon 2 and 2.4-kb of intron 2 [57]. The data show that in epithelial (HEK 293) and osteoblast (HOS) cell lines the inserted neo cassette had no effect on P2 activity whereas in T-cell (Jurkat), myeloid (U937) and neuronal (PC12) cell lines the neo cassette significantly attenuated the activity of Runx1 P2. [file 1471-213X-7-84-S1.pdf]

Additional file 1

Insertion of a neo cassette into Runx1 P2 region inhibits transcriptional activity in transfected cells.

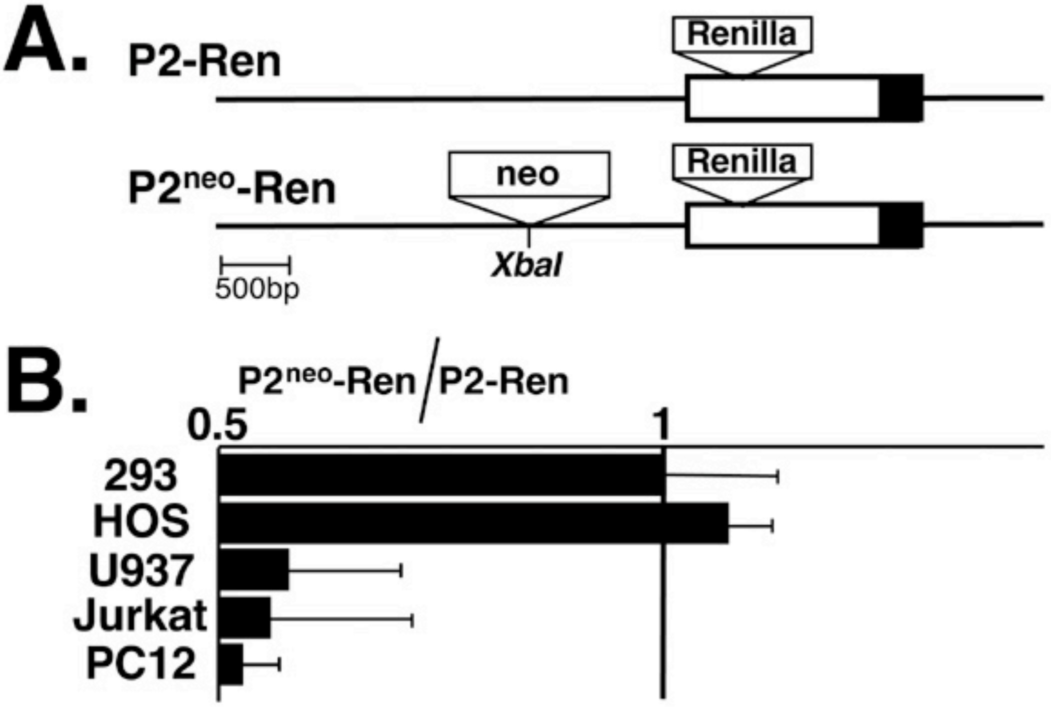

Two reporter plasmids were constructed (designated P2-Ren and P2^{neo}-Ren) in which a *Runx1* genomic region spanning 3Kb upstream of P2 transcription start sites regulated the expression of Renilla luciferase gene. **(A)** Schematic drawing of P2-Ren and P2^{neo}-Ren reporter plasmids. Both contained a genomic region which spans 3-kb upstream of P2 transcriptional start site (TSS), complete exon 2 and 2.4-kb of intron 2 [1]. Renilla luciferase gene was placed in exon 2 inside the P2-5'UTR, 1.2-kb upstream of the ATG codon. The P2^{neo}-Ren construct contains a neo cassette inserted into the Xba I site at nucleotide 27675861 of the mouse genome (accession number NT-039625), which is located 1-kb upstream of P2 TSS. **(B)** P2 activity was assessed by transfection P2-Ren and P2^{neo}-Ren into five different cell lines. CMV-luciferase plasmid was co-transfected to monitor transfection efficiency. 48h post transfection, enzymatic activities of renilla and luciferase were determined and renilla/luciferase ratio calculated. Results are presented as renilla/luciferase ratio of triplicated transfection assays of P2^{neo}-Ren relative to the renilla/luciferase ratio obtained in the same experiment with P2-Ren transfected cells. The bars represent the average ±S.E. of three independent experiments. The data show that in epithelial (HEK 293) and osteoblast (HOS) cell lines the

inserted neo cassette had no effect on P2 activity whereas in T-cell (Jurkat), myeloid (U937) and neuronal (PC12) cell lines the neo cassette significantly attenuated the activity of *Runx1* P2.

1. D Levanon, G Glusman, T Bangsow, E Ben-Asher, DA Male, N Avidan, C Bangsow, M Hattori, TD Taylor, S Taudien, et al: **Architecture and anatomy of the genomic locus encoding the human leukemia-associated transcription factor RUNX1/AML1**. Gene 2001, **262**:23-33.

Methods

Cell cultures and transfections

Cell lines 293 (human embryonic kidney epithelial) and HOS (human osteosacoma), were maintained in Dulbeco's modified Eagle's medium (DMEM) (GibcoBRL) supplemented with 10% V/V of new born calf serum (NCS) (GibcoBRL), 2mM L-glutamine, 100unit/ml penicillin and 10mg/ml streptomycin. U937 (pro-monocytes) and Jurkat (human T lymphocytes) were grown in RPMI-1640 medium (SIGMA) supplemented with 10% NCS and antibiotics as before. PC12 cells (rat neuronal pheochromocytoma) were grown in DMEM supplemented with 8% heat inactivated (HI) NCS, 8% HI horse serum (SIGMA), L-glutamine and antibiotics. Plasmid DNAs were prepared using the CONCERT plasmid purification kit (GibcoBRL). Exponentially growing 293 and HOS cells were transfected with plasmid DNA using the standard method of $CaPO_4$-mediated transfection followed by incubation for 48 hrs at 37^0C prior to analysis. Electroporation was employed for the hematopoietic cell lines. Cells (1.5×10^7) in log phase were transfected as specified by the manufacturer (BTX), using a capacitor discharge of 250V, 1700mF, and 13Ω (R1) for U937 cells, or 250V, 1700mF, and 72Ω (R4) for Jurkat. Cells were electroporated with addition of salmon sperm DNA and 2xHeBS buffer. Following electroporation, the cells were immediately transferred into 18ml of growth medium and incubated for 48 hrs prior to analysis. PC12 cells (2.4×10^4) were transfected by Lipofectamin reagent (Invitrogen) according to manufacturer's instructions. Equal molar amounts of P2-Ren and $P2^{neo}$-Ren plasmids were employed. Each transfection included CMV-luciferase plasmid as an internal control for transfection efficiency. Cell extracts were collected and assayed for luciferase and Renilla activities using Dual Luciferase Reporter Assay System according to manufacturer (Promega) instructions, with a TD-200 luminometer (Turner).
