# Supplementary material for: Developmentally regulated promoter-switch transcriptionally controls Runx1 function during embryonic hematopoiesis
Source: BMC Dev Biol. 2007 Jul 12;7:84. doi: 10.1186/1471-213X-7-84 (PMC1941738; doi:10.1186/1471-213X-7-84)
Supplement: Additional file 2 — Generation of Runx1 P2neo/neo mice and phenotypic analysis of mutant newborns. (A, B) Scheme of genomic organization of Runx1 outlining the steps employed to generate the mutant P2neo locus and Southern blot analysis of genomic DNA identifying homologous recombination in ES clones and in newborn mutant mice. The region within P2-5'UTR (accession # D26532), which was used as a probe for in-situ hybridization is indicated on the targeting construct (striped bars), whereas the primers used for RT-PCR analysis are indicated on the genomic scheme (arrow heads). F1 heterozygotes Runx1P2neo (P2neo) were intercrossed and all three genotypes were detected in F2 litters. Transmission of the mutant allele roughly followed a Mendelian inheritance pattern, indicating that mice homozygous for the Runx1 mutant allele were born. The neo gene was excised (Mutant locus->Neo minus locus) by crossing heterozygous Runx1 P2neo/+ mice onto the appropriate Cre transgenic mice as described in results. (C) Early neonatal lethality of homozygous Runx1 P2neo/neo mice. P2neo/neo neonates exhibit marked growth retardation and die within few days after birth. At birth mutant mice were as active as their littermates, exhibited suckling behavior and had milk in their stomachs. However, at day 2 the amount of milk in the stomach of P2neo/neo mice drastically decreased. (D) Body weights of newborn WT and P2neo/neo mice during the first three days as observed in two litters (n = 14). Newborns were weighed at the indicated time after birth. WT and P2neo/+ mice gained weight, whereas P2neo/neo did not. (E) Stomach and duodenum of P2.5 WT and P2neo/neo littermate mice. Volume of milk in P2neo/neo stomach was significantly lower compared to WT. To further characterize the phenotype/genotype relationships in P2neo/neo mice, the neo gene was removed, as described in the results and shown in (B). As removal of the neo gene rescued the early lethality phenotype of P2neo/neo newborns, we concluded that the P2n [file 1471-213X-7-84-S2.pdf]

Additional File 2

Generation of *Runx1* P2$^{neo/neo}$ mice and phenotypic analysis of mutant newborns.

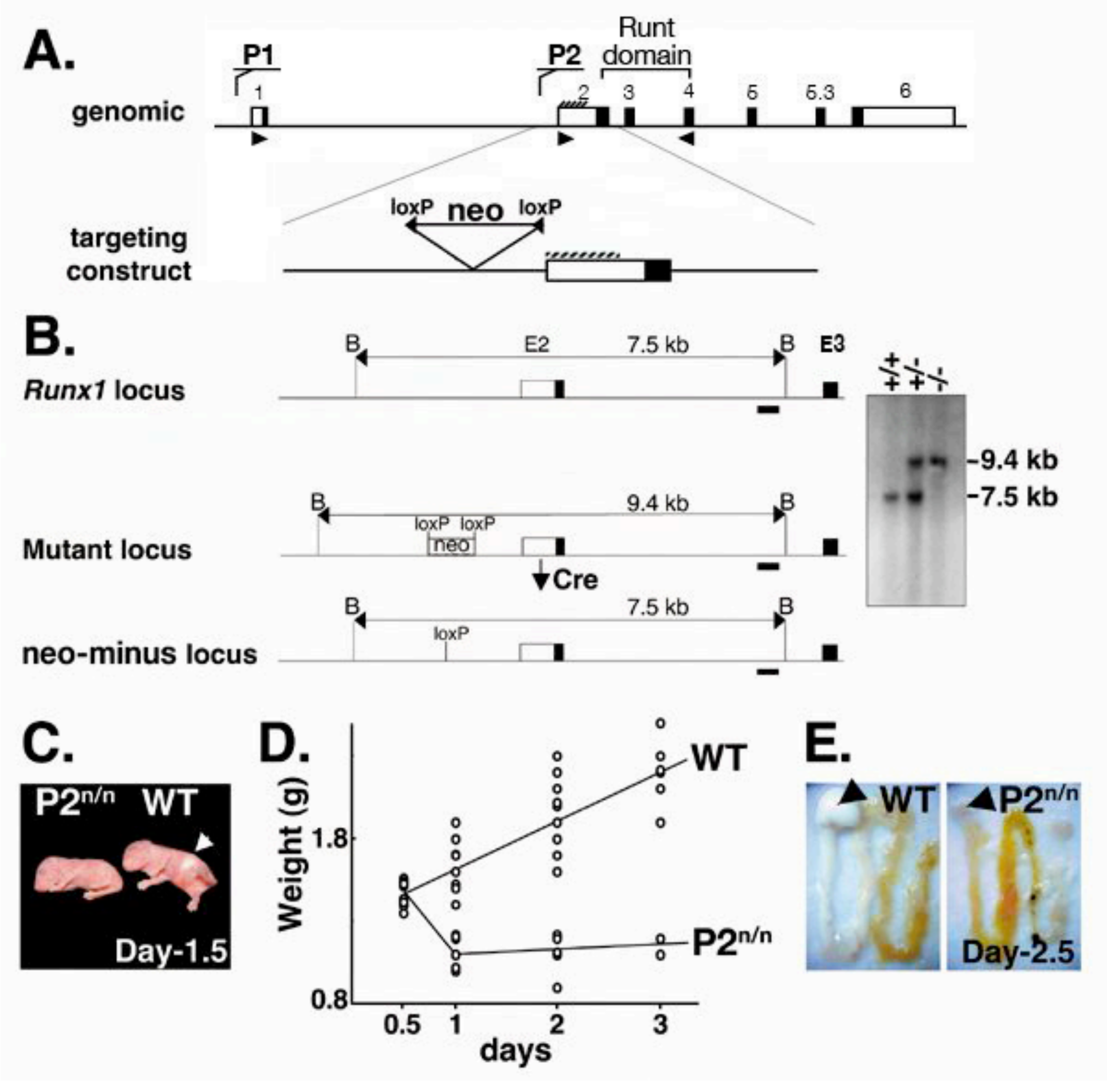

(A) Scheme of genomic organization of *Runx1.* Coding exons are presented in black and non-coding UTRs in white. To generate the targeting construct, a lox-P-flanked *neo* gene driven by the *pgk* promoter was inserted into the P2 *XbaI* site indicated in figure S1. The targeting construct spans a 7.2Kb *EcoRI-PstI* genomic fragment and was flanked by a 1.86Kb at the 5′ and 5.35Kb at the 3′. The region within P2-5′UTR which was used as a probe for *in-situ* hybridization is indicated on the targeting construct (striped bars), whereas the primers used for RT-PCR analysis are indicated on the genomic scheme (arrow heads). **(B)** Schematic representation of the steps generating the mutant locus and Southern blot analysis of genomic DNA identifying homologous recombination in ES clones and in newborn mutant mice. Left part, a probe from outside the recombination locus (horizontal black bar), was used to screen for

homologous recombination. The positions of *Bam*HI restriction enzyme cutting sites [B] and exon numbers (Levanon 2001 gene) are indicated. Right part, Southern blot analysis of genomic DNA from three newborn mice. The probe hybridizes to a 7.5- and a 9.4-Kb *Bam*HI fragment derived from WT and the targeted *Runx1* allele, respectively. F1 heterozygotes *Runx1*^{P2neo} (P2^{neo}) were intercrossed and all three genotypes were detected in F2 litters. Transmission of the mutant allele roughly followed a Mendelian inheritance pattern, indicating that mice homozygous for the *Runx1* mutant allele were born. The *neo* gene was excised (Mutant locus->Neo minus locus) by crossing heterozygous *Runx1* P2$^{neo/+}$ mice onto the appropriate *Cre* transgenic mice as described in results. **(C)** Early neonatal lethality of homozygous *Runx1* P2$^{neo/neo}$ mice. P2$^{neo/neo}$ neonates exhibited marked growth retardation and die within few days after birth. At birth mutant mice were as active as their littermates, exhibiting suckling behavior and had milk in their stomachs. However, at day 2 the amount of milk in the stomach of P2$^{neo/neo}$ mice drastically decreased. Side view of WT and P2$^{neo/neo}$ littermates at P1.5. P2$^{neo/neo}$ newborns were malnourished and dehydrated as also evidenced by the lower amount of milk in their stomach (arrow). **(D)** Body weights of newborn WT and P2$^{neo/neo}$ mice during the first three days as observed in two litters (n=14). Newborns were weighed at the indicated time after birth. WT and P2$^{neo/+}$ mice gained weight, wherease P2$^{neo/neo}$ did not. The number of viable P2$^{neo/neo}$ pups declined and in these particular litters none survived beyond P3. **(E)** Stomach and duodenum of P2.5 WT and P2$^{neo/neo}$ littermate mice. Volume of milk in P2$^{neo/neo}$ stomach (arrow) was significantly lower compared to WT. To further characterize the phenotype/genotype relationships in P2$^{neo/neo}$ mice, the *neo* gene was removed, as described in the results and shown above in (B). As removal of the *neo* gene rescued the early lethality phenotype of P2$^{neo/neo}$ newborns, we concluded that the P2$^{neo/neo}$ phenotype resulted from the presence of *neo* in the P2 region.

As the analysis of the volume of milk in the stomach clearly indicated that P2$^{neo/neo}$ newborns were malnourished (2C and 2E) the P2$^{neo/neo}$ newborns were further examined. Suckling in neonatal mice involves both coordinated rhythmic mouth movements and swallowing. Like control littermates, P2$^{neo/neo}$ pups demonstrated rhythmic jaw movements upon manual stimulation and had no gross facial structural defects. However, they failed to thrive even when conditions favoring their access to feeding where created by removing most of the WT littermates. We thus concluded that the diminished feeding behavior of P2$^{neo/neo}$ mice was not due to physical disability or to a failure of the mice to compete with the fitter WT littermates. Additionally, P2$^{neo/neo}$ neonates were always observed in the same group with WT littermates under the nursing

mother, indicating that they were not selectively ignored. Thus, while the above observations indicated that P2$^{neo/neo}$ newborns were malnourished, no obvious explanation for the neonatal lethality was apparent.
